# Urolithin A activates mitophagy via the AMPK–mTOR axis and modulates the gut–ceramide axis to ameliorate cardiac remodeling in HFpEF

**DOI:** 10.1038/s12276-026-01776-2

**Published:** 2026-07-10

**Authors:** Hangyul Song, Chahyeon Yun, Yunju Choi, Wooju Jeong, Yumin Kim, Jaeyoung Kim, Ju-Yeon Lee, Dongryeol Ryu, Sang-Wook Park, Chang-Myung Oh

**Affiliations:** 1 Department of Biomedical Science and Engineering, Gwangju Institute of Science and Technology, Gwangju, South Korea; 2 Department of Dental Bioscience, School of Dentistry, Chonnam National University, Gwangju, South Korea; 3 Prestigebiopharma Innovate Discovery Center, Busan, South Korea

**Keywords:** Heart failure, Therapeutics

## Abstract

Heart failure with preserved ejection fraction (HFpEF) accounts for nearly half of all heart failure cases. However, effective therapies targeting its underlying pathophysiological mechanisms remain lacking. Previous studies have indicated mitochondrial dysfunction and impaired mitophagy as key contributors to HFpEF pathophysiology. In this study, we investigated whether urolithin A (UA), a gut microbiome-derived mitophagy-activating compound, can ameliorate HFpEF. A two-hit mouse model was established using a high-fat diet and Nω-nitro-L-arginine methyl ester, and UA was administered during disease progression. In vitro and in vivo experiments, together with multi-omics analyses, showed that UA alleviated diastolic dysfunction, cardiac hypertrophy, and fibrosis in HFpEF mice. These effects were accompanied by restoration of mitochondrial ultrastructure and enhanced mitochondrial respiration and glycolytic capacity. Notably, UA activated AMPK signaling while inhibiting mTOR, promoting ULK1-dependent autophagy initiation and restoring impaired mitophagic flux. These effects were associated with improved mitochondrial quality control and function. Concurrently, multi-omics analyses revealed that UA remodels the gut microbiome–ceramide axis and reduces circulating ceramide accumulation, thereby alleviating lipotoxic stress. Furthermore, single-nucleus transcriptomic analysis revealed that UA treatment leads to the attenuation of fibrosis-related cellular programming in human induced pluripotent stem cell-derived cardiomyocytes. Taken together, these findings indicate that UA improves cardiac remodeling in HFpEF by activating mitophagy-dependent mitochondrial quality control and modulating the gut microbiome–ceramide axis, highlighting its potential as a mechanism-based, mitochondria-targeted therapeutic strategy for HFpEF.

## Urolithin A enhances mitochondrial function in heart failure

Heart failure with preserved ejection fraction (HFpEF) is a complex syndrome with limited treatment options, often linked to mitochondrial dysfunction. This study explores the potential of urolithin A (UA), a metabolite known to enhance mitophagy, in addressing HFpEF. Researchers used a two-hit mouse model combining a high-fat diet and nitric oxide synthase inhibition to mimic HFpEF conditions. They administered UA and used multi-omics techniques to analyze its effects. Results showed that UA improved cardiac function, reduced fibrosis, and enhanced mitochondrial quality control by activating AMPK signaling and suppressing mTOR activity. UA also modulated the gut microbiome, reducing ceramide levels, which are linked to cardiac stress. These findings suggest UA’s potential as a therapeutic strategy for HFpEF, highlighting the need for further research to confirm its efficacy and safety in humans.

This summary was initially drafted using artificial intelligence, then revised and fact-checked by the author.

## Introduction

Heart failure with preserved ejection fraction (HFpEF) accounts for approximately half of all heart failure cases and represents a major unmet clinical need worldwide^1^. HFpEF is a heterogeneous syndrome driven by multiple comorbidities, including obesity, hypertension, and diabetes, which converge to induce systemic inflammation, endothelial dysfunction, and myocardial remodeling. Although sodium-glucose cotransporter-2 inhibitors have recently shown modest clinical benefits, mechanism-based therapies addressing the fundamental cellular pathology of HFpEF remain absent^2,3^.

Mitochondrial dysfunction has emerged as a central pathogenic mechanism in HFpEF. The enormous energy demand of the heart necessitates efficient oxidative phosphorylation and robust mitochondrial quality control (MQC) to sustain the contractile and relaxation functions. In HFpEF, this homeostasis is disrupted, leading to the accumulation of damaged mitochondria, which generate excessive reactive oxygen species, impair ATP production, and dysregulate calcium handling^4,5^. A critical component of MQC is mitophagy, the selective autophagic removal of dysfunctional organelles^6^. Defective mitophagy has been implicated in age-related cardiovascular diseases and metabolic cardiomyopathy, suggesting that the restoration of this clearance mechanism represents a rational therapeutic strategy^6–8^. However, no clinically approved mitophagy-enhancing therapies exist for HFpEF, and whether such approaches can reverse the established pathology of this complex syndrome remains to be determined.

Urolithin A (UA) is a gut microbiome-derived metabolite produced from dietary ellagitannins found in pomegranates, berries, and walnuts^9^. UA activates mitophagy across species ranging from *Caenorhabditis* *elegans* to humans, improving muscle function and extending the healthspan^10–12^. Although preclinical studies have demonstrated cardioprotective effects in ischemia–reperfusion and diabetic cardiomyopathy models^13,14^, the therapeutic efficacy of UA in HFpEF, in which metabolic stress and structural remodeling predominate, has not yet been investigated. Here, we investigated the therapeutic potential of UA in a two-hit mouse model of HFpEF that combined a high-fat diet (HFD) and nitric oxide synthase inhibition to recapitulate the key metabolic and hemodynamic features of the human disease^15,16^. We hypothesized that UA would ameliorate diastolic dysfunction by restoring mitophagy and improving mitochondrial homeostasis. Using an integrated multiomics approach encompassing shotgun metagenomics, plasma lipidomics, and single-nucleus RNA-sequencing, we delineated systemic and cell-autonomous mechanisms linking mitochondrial restoration to the suppression of profibrotic and inflammatory programs. Our findings demonstrated that UA ameliorated HFpEF pathology in this preclinical model through the coordinated modulation of mitophagy, ceramide metabolism, and fibrogenic programs and provided a mechanistic rationale for its therapeutic development in HFpEF.

## Materials and methods

### Cell cultures

H9c2 rat cardiomyoblasts were maintained in Dulbecco’s modified Eagle’s medium (Gibco) supplemented with 10% fetal bovine serum and cultured under standard conditions (37 °C, 5% CO_2_). Cells were exposed to control conditions or to HF-like stress conditions induced by combined palmitate and Nω-nitro-L-arginine methyl ester (L-NAME) treatment. Where indicated, cells were treated with UA for 24 h before downstream molecular, imaging, and functional assays. UA was dissolved in dimethyl sulfoxide (DMSO) to generate a 10 mM stock solution, aliquoted, and stored at −20 °C. For experiments, UA was diluted in culture medium to a final concentration of 10 μM, with the same final concentration of DMSO applied to control conditions. Human induced pluripotent stem cells (hiPSCs) were maintained on Matrigel-coated plates and differentiated into cardiomyocytes (CMs) using a chemically defined cardiac induction protocol. Spontaneous beating was observed between days 8 and 10 of differentiation, and experiments were conducted on days 15–20. Where enrichment was required, CMs were purified using minus-insulin metabolic selection.

### Mouse experiments

All animal experiments were approved by the Institutional Animal Care and Use Committee of Gwangju Institute of Science and Technology (GIST-2021-110) and conducted in accordance with NIH and ARRIVE guidelines. Eight-week-old male C57BL/6J mice were randomly assigned to control, HFpEF, or HFpEF + UA groups. HFpEF was induced using a two-hit protocol consisting of a HFD (60% kcal from fat) combined with L-NAME administration in drinking water. Control mice received standard chow and water. UA was administered by mixing into the HFD at doses of 250 mg/kg/day or 500 mg/kg/day throughout the experimental period. Body weight was monitored weekly^17–19^.

### Echocardiography

Transthoracic echocardiography was performed using small-animal ultrasound systems (VINNO 6 LAB or VisualSonics Vevo 2100) under light isoflurane anesthesia. Left ventricular systolic function was assessed from mid-ventricular short-axis M-mode images by measuring ejection fraction and fractional shortening. Diastolic function was evaluated from apical four-chamber views using pulsed-wave and tissue Doppler imaging to quantify transmitral E and A waves. Measurements were performed in accordance with established guidelines^20–22^.

### Histological analysis

Hearts were fixed in 4% paraformaldehyde, paraffin-embedded, and sectioned at 6 μm. Cardiac morphology and fibrosis were assessed by hematoxylin and eosin and Masson’s trichrome staining, respectively. Fibrotic area was quantified using ImageJ/Fiji under blinded conditions.

### Transmission electron microscopy

For ultrastructural analysis, fixed cardiac tissues were processed for transmission electron microscopy. Ultrathin sections were imaged at high magnification, and mitochondrial number, size, and structural integrity were quantified from multiple randomly selected fields per sample^23–25^.

### Immunoblotting and quantitative RT-PCR

Protein extracts from cells or tissues were resolved by SDS–PAGE and subjected to immunoblotting for autophagy/mitophagy and signaling markers, including ULK1, PINK1, Parkin, LC3, p62, AMPK, mTOR, and AKT. Band intensities were quantified by densitometry and normalized to β-actin. Total RNA was isolated, reverse-transcribed, and analyzed by quantitative real-time PCR using SYBR Green chemistry. Relative gene expression was calculated using the ΔΔ*C*_t_ method and normalized to housekeeping genes. Primer sequences are provided in Supplementary Table 1.

### Seahorse mitochondrial stress test and mitophagy assays

Mitochondrial respiration was measured using the Seahorse XF Mito Stress Test. Basal respiration, ATP-linked respiration, maximal respiration, and spare respiratory capacity were calculated, with oxygen consumption rates normalized to total protein content. Mitophagy was assessed using the mt-Keima reporter in H9c2 CMs. Ratiometric fluorescence imaging was performed under identical acquisition settings, and mitophagy was quantified as the per-cell 561/458 nm fluorescence ratio. Carbonyl cyanide *m*-chlorophenyl hydrazone was used as a positive control.

### Bulk-sequencing (RNA-seq)

Sequencing data were processed using Python-based pipelines to obtain normalized expression values. Differentially expressed genes were identified using Wald tests, with effect sizes summarized as log_2_ fold changes. *P*-values were integrated using Stouffer’s method, followed by false discovery rate (FDR) correction. Genes meeting significance criteria were subjected to Gene Ontology and Gene Set Enrichment Analysis^26–28^.

### Lipidomics

Plasma lipidomic profiling was performed using the MxP Quant 500XL kit (Biocrates Life Sciences AG), following the manufacturer’s standardized protocol. Whole blood was collected from the abdominal vena cava into heparinized tubes, maintained on ice, and centrifuged at 2,000×*g* for 15 min at 4 °C. Plasma was aliquoted and stored at −80 °C until analysis. Samples were prepared in 96-well plates including multilevel calibration standards, three QC levels, and blanks per plate. Amino acids and biogenic amines underwent PITC derivatization, whereas lipid species were analyzed without derivatization. Liquid chromatography–tandem mass spectrometry was performed on a Triple Quad 5500+ mass spectrometer coupled to an ACQUITY UPLC system, whereas lipid species and hexoses were quantified by flow injection analysis. Data were processed using the Biocrates WebIDQ/MetIDQ platform with internal standards and matrix QCs applied. Analytes failing vendor-defined quality criteria were excluded a priori from downstream analyses^29^.

### Shotgun metagenomic sequencing

Fecal samples were collected using sterile forceps and stored at −80 °C. Genomic DNA was extracted using a bead-beating-based lysis protocol optimized for Gram-positive bacteria, followed by column-based purification. Libraries were sequenced to a depth of approximately 44 million paired-end reads per sample. After quality filtering and removal of host-derived reads by alignment to the mouse reference genome, taxonomic profiling was performed using MetaPhlAn v3.0. Functional profiling was conducted using HUMAnN v3.0 against the UniRef90 database, with gene families regrouped to Kyoto Encyclopedia of Genes and Genomes orthologs. Alpha diversity was assessed using the Shannon index, and beta diversity was quantified using Bray–Curtis dissimilarity. Differential taxa were identified using linear discriminant analysis effect size with FDR correction^30–32^.

Single-nucleus RNA-sequencing was performed on hiPSC-derived CMs under control, HL, and UA-treated conditions using the 10× Genomics Chromium platform. Libraries were sequenced on an Illumina NovaSeq 6000 and processed with Cell Ranger v8.0.1. Gene–barcode matrices were analyzed in Seurat v5. Low-quality nuclei were excluded based on gene counts, UMI counts, and mitochondrial and ribosomal read fractions. Data were normalized using SCTransform and integrated across conditions. Clustering was performed using a shared nearest neighbor graph, and visualization was carried out using uniform manifold approximation and projection (UMAP). Differential gene expressions (DGEs) were assessed using both single-nucleus Wilcoxon tests and pseudobulk DESeq2 analyses. CM subpopulations were identified based on canonical markers, and state transitions were inferred using pseudotime trajectory analysis implemented in scFates.

### Statistical analysis

Data are presented as mean ± standard error of the mean. Group comparisons were performed using two-way analysis of variance with Dunnett’s post hoc test or Kruskal–Wallis test with Dunn’s correction, as appropriate. Transcriptomic analyses used Wald tests with FDR correction, and *P*-values from multiple tests were combined using Stouffer’s method. Statistical significance was defined as *P* < 0.05 or FDR < 0.05.

## Results

### UA treatment restored diastolic function in a two-hit HFpEF mouse model

**Fig. 1:**
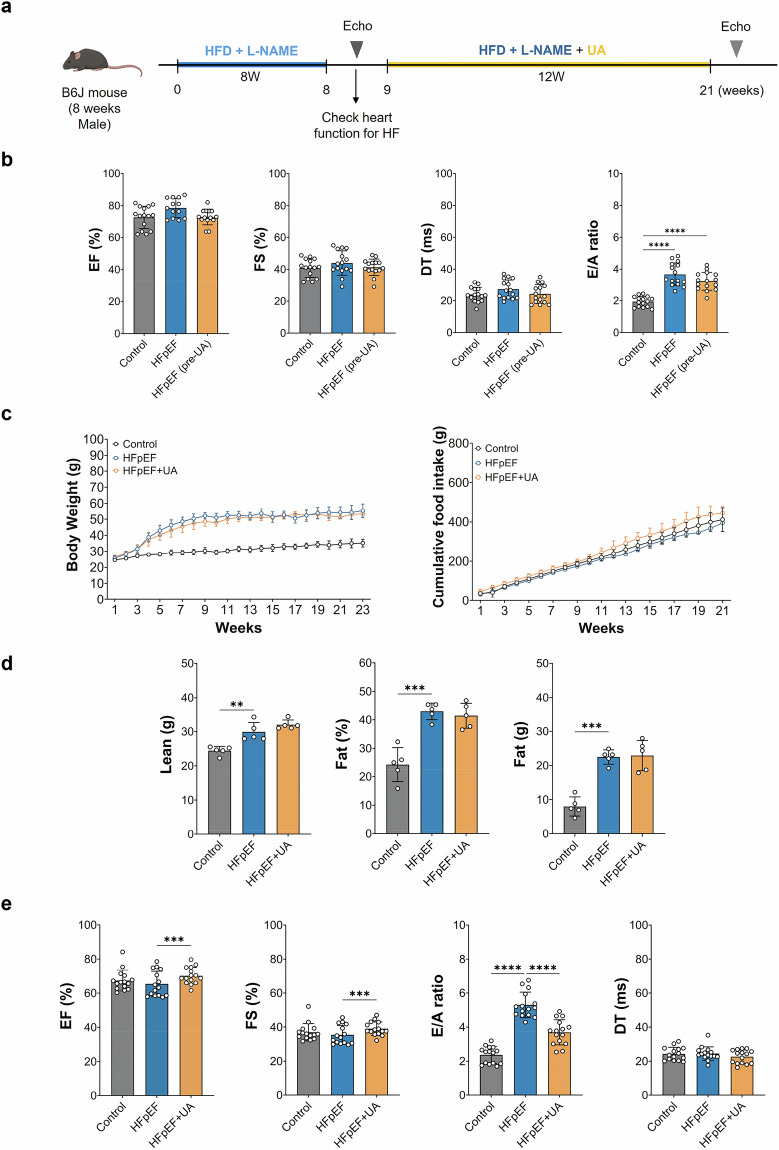
Experimental design and physiological characterization of a two-hit HFpEF mouse model with UA intervention. **a** Schematic overview of the experimental timeline. Eight-week-old male C57BL/6J mice were subjected to a two-hit heart failure with preserved ejection fraction (HFpEF) protocol consisting of high-fat diet (HFD) and Nω-nitro-L-arginine methyl ester (L-NAME) administration for 8 weeks, followed by an additional 12 weeks of continued HFD and L-NAME with or without urolithin A (UA) treatment. Echocardiographic assessments were performed at weeks 8 and 21. **b** Echocardiographic evaluation of systolic and diastolic function before UA treatment (week 8), including ejection fraction (EF), fractional shortening (FS), *E*/*A* ratio, and deceleration time (DT) (*n* = 15 mice per group). **c** Longitudinal measurements of body weight and cumulative food intake over the study period in control, HFpEF, and HFpEF+UA groups (*n* = 15 mice per group). **d** Endpoint body composition analysis showing lean mass, fat percentage, and fat mass at study termination (*n* = 5 mice per group). **e** Echocardiographic assessment at week 21 demonstrating systolic and diastolic functional parameters following UA treatment (*n* = 15 mice per group). All data are presented as mean ± SEM. *n* represents the number of biologically independent animals. Exact sample sizes for each analysis are indicated in the corresponding panels. Statistical significance was assessed using one-way or two-way analysis of variance with Dunnett’s multiple comparisons test, or the Kruskal–Wallis test with Dunn’s post hoc test, as appropriate. *P < 0.05, **P < 0.01, ***P < 0.001.

To model the complex pathophysiology of HFpEF, 8-week-old male C57BL/6J mice were subjected to a two-hit protocol that combined HFD and L-NAME administration for 8 weeks (Fig. 1a). Echocardiography at week 8 confirmed successful induction of the HFpEF phenotype; the ejection fraction remained within the normal range; however, the diastolic function was markedly impaired, as demonstrated by a significantly elevated E/A ratio, indicative of restrictive filling (Fig. 1b). After phenotypic confirmation, the mice continued the two-hit regimen with the co-administration of UA for an additional 12 weeks. Notably, UA treatment had no effect on the body weight trajectories, daily food consumption, or body composition parameters, including lean and fat masses (Fig. 1c, d), indicating that the metabolic effects of the HFpEF-inducing regimen were not altered by UA and suggesting metabolic tolerability.

Terminal echocardiographic assessment at week 20 revealed significant functional improvements in the UA-treated mice. Both systolic parameters (ejection fraction and fractional shortening) were increased compared with those in untreated HFpEF mice. Furthermore, the diastolic function markedly improved, with the *E*/*A* ratio decreasing toward the control values (Fig. 1e), consistent with restored ventricular relaxation and filling dynamics. Collectively, these data demonstrated that UA treatment ameliorated cardiac dysfunction in HFpEF without confounding metabolic alterations.

### UA treatment attenuated pathological cardiac hypertrophy and fibrosis in HFpEF mice

**Fig. 2:**
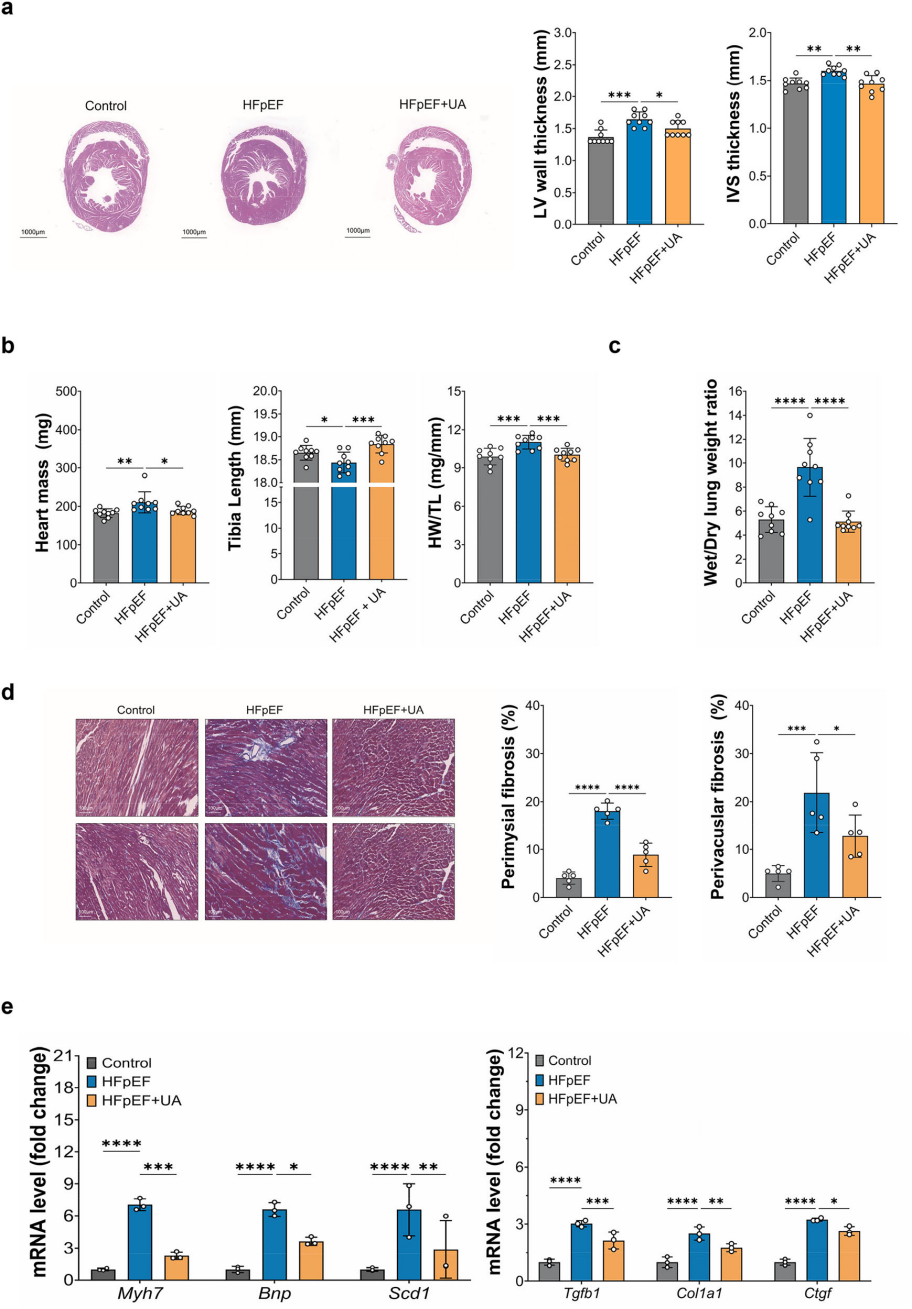
Structural and molecular characterization of cardiac remodeling in a two-hit HFpEF mouse model. **a** Representative hematoxylin and eosin-stained short-axis cardiac sections from control, heart failure with preserved ejection fraction (HFpEF), and HFpEF+UA mice, shown from left to right. Quantification of left ventricular (LV) wall thickness and interventricular septal (IVS) thickness is shown on the right (*n* = 9 mice per group). Scale bar, 1000 µm. **b** Gravimetric and anatomical measurements, including heart mass, tibia length, and heart weight-to-tibia length ratio (HW/TL) (*n* = 9 mice per group). **c** Lung wet-to-dry weight ratio measured at the study end point as an index of pulmonary congestion (*n* = 9 mice per group). **d** Representative Masson’s trichrome-stained LV sections illustrating myocardial collagen deposition across experimental groups. Quantification of perimysial (left) and perivascular (right) fibrosis is shown on the right (*n* = 5 mice per group). Scale bar, 100 µm. **e** Quantitative PCR analysis of hypertrophic/metabolic stress-related markers (Myh7, Bnp, and Scd1) and fibrosis-associated genes (Tgfb1, Col1a1, and Ctgf) in heart tissue (*n* = 3). Gene expression levels were normalized to internal controls and expressed relative to control. All data are presented as mean ± SEM. *n* represents the number of biologically independent animals. Exact sample sizes for each analysis are indicated in the corresponding panels. Statistical significance was assessed using one-way analysis of variance followed by Dunnett’s multiple comparisons test or the Kruskal–Wallis test followed by Dunn’s post hoc test, as indicated. *P < 0.05, **P < 0.01, ***P < 0.001. UA, urolithin A.

To examine the structural basis of diastolic dysfunction in HFpEF, we evaluated whether UA treatment could reverse the pathological remodeling at the organ and cellular levels. Hematoxylin and eosin staining of short-axis cardiac sections revealed concentric left ventricular hypertrophy with thickened walls in HFpEF mice (Fig. 2b). This was confirmed by gravimetric analysis; the heart weight-to-tibia length ratio increased in HFpEF mice compared with controls, and the effect was attenuated by UA treatment (Fig. 2b). HFpEF mice developed pulmonary congestion because of elevated filling pressures, as evidenced by the increased lung weight-to-tibia length ratio (Fig. 2c). UA treatment normalized this parameter, consistent with the improved *E*/*A* ratio observed on echocardiography (Fig. 1e).

Myocardial fibrosis, a hallmark of diastolic stiffness in HFpEF, was evaluated using Masson’s trichrome staining (Fig. 2d). HFpEF hearts exhibited extensive collagen deposition in both interstitial and perivascular compartments, whereas UA treatment markedly reduced the extracellular matrix (ECM) accumulation (Fig. 2d). At the molecular level, fibrosis-associated genes were significantly upregulated in the HFpEF myocardium, including transforming growth factor beta 1 (*Tgfb1*), collagen type 1 alpha 1 (*Col1a1*), and connective tissue growth factor (*Ctgf*). UA treatment attenuated these transcriptional changes and restored the expression levels to control values (Fig. 2e). Similarly, the hypertrophic gene markers including myosin heavy chain 7 (*Myh7*) and natriuretic peptide B (*Nppb*) were elevated in the HFpEF group and normalized by UA treatment (Fig. 2e). These histological and molecular findings demonstrated that UA mitigated the maladaptive structural remodeling in HFpEF, thereby providing a mechanistic basis for the preservation of diastolic function.

### UA treatment activates AMPK–mTOR signaling and restores MQC in HFpEF

**Fig. 3:**
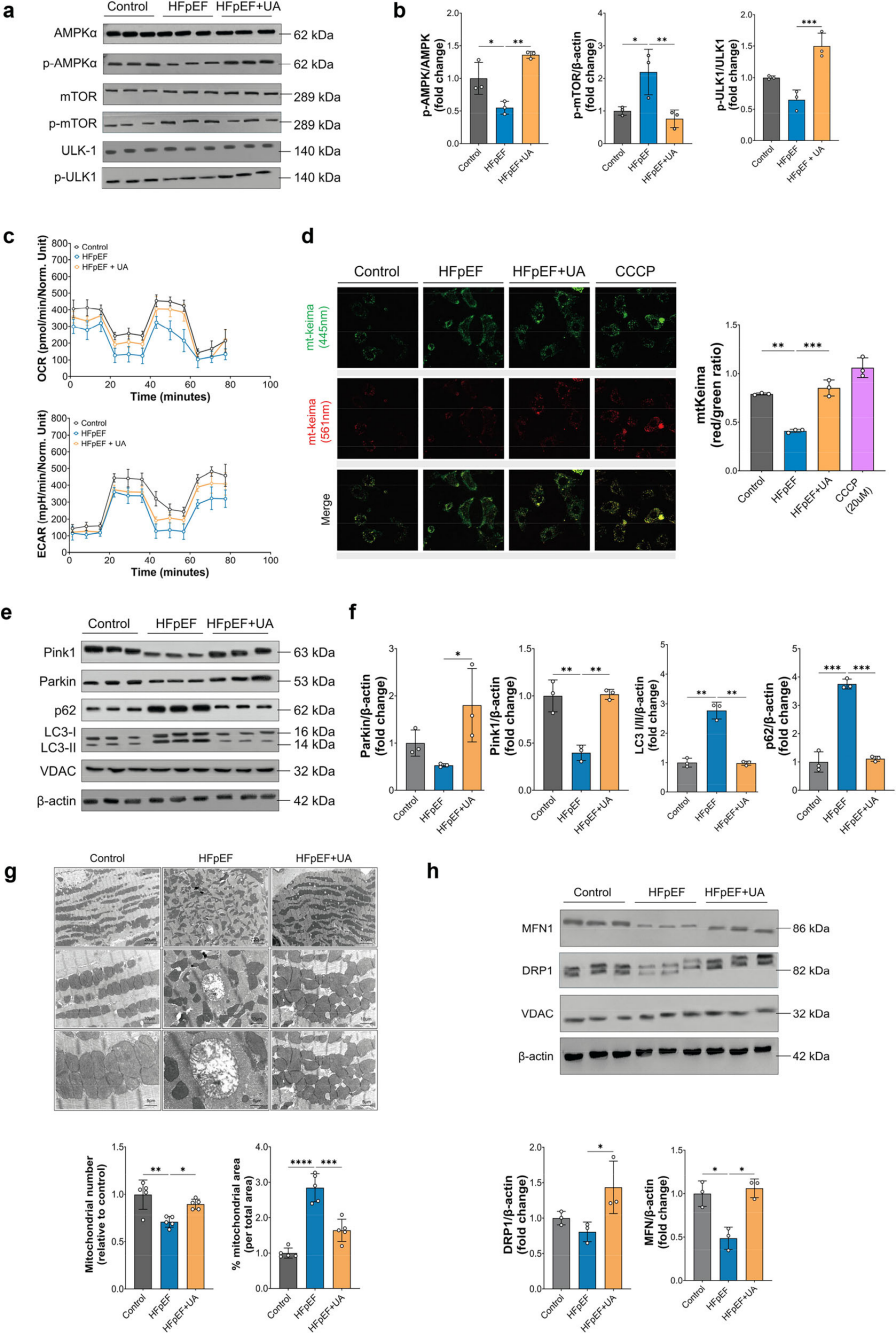
Mitochondrial function and mitophagy-related signaling in HFpEF hearts following UA treatment. **a** Immunoblot analysis of key regulators of the AMPK–mTOR–ULK1 signaling axis, including AMPKα and phospho-AMPKα, mTOR and phospho-mTOR, and ULK1 and phospho-ULK1, in cardiac tissue lysates from control, heart failure with preserved ejection fraction (HFpEF), and HFpEF+UA mice. **b** Densitometric quantification of p-AMPK/AMPK, p-mTOR/mTOR, and p-ULK1/ULK1 ratios normalized to total protein levels (*n* = 3). **c** Seahorse extracellular flux analysis showing oxygen consumption rate (OCR) and extracellular acidification rate (ECAR) under basal and stress conditions (*n* = 3 independent experiments). **d** Representative confocal microscopy images of mt-Keima-expressing H9c2 cells showing mitochondrial localization at neutral (green, 458 nm) and acidic (red, 561 nm) pH conditions, indicating mitophagy flux. Carbonyl cyanide *m*-chlorophenyl hydrazone (CCCP) was used as a positive control. Quantification of the mt-Keima red/green fluorescence ratio is shown on the right (*n* = 3 independent experiments). **e** Immunoblot analysis of mitophagy-related proteins in cardiac tissue lysates, including PINK1, Parkin, p62/SQSTM1, LC3-I/II, VDAC, and β-actin. **f** Densitometric quantification of Parkin, PINK1, LC3-II, and p62 levels normalized to β-actin (*n* = 3). **g** Representative transmission electron microscopic images of cardiac tissue showing mitochondrial ultrastructure across control, HFpEF, and HFpEF+UA groups. Quantification of mitochondrial number (relative to control) and mitochondrial area (% of total tissue area) is shown below (*n* = 5 mice per group). **h** Immunoblot analysis of mitochondrial dynamics proteins DRP1 and MFN1, together with VDAC and β-actin, with corresponding densitometric quantification of DRP1 and MFN1 shown below (*n* = 3). All data are presented as mean ± SEM. For in vivo experiments, *n* represents the number of biologically independent animals. For in vitro experiments, *n* represents the number of independent experiments (biological replicates). Exact sample sizes for each analysis are indicated in the corresponding panels. Statistical significance was assessed using one-way analysis of variance followed by Dunnett’s multiple comparisons test or the Kruskal–Wallis test followed by Dunn’s post hoc test, as indicated. *P < 0.05, **P < 0.01, ***P < 0.001. Norm., normalized; UA, urolithin A; VDAC, voltage-dependent anion channel.

MQC is essential for maintaining cardiac bioenergetics under pathological stress^7^. To elucidate the mechanistic basis of UA-mediated cardioprotection, we first examined key signaling pathways regulating mitophagy^33^. Immunoblot analysis revealed that HFpEF hearts exhibited reduced AMPK phosphorylation and increased mTOR activation, indicating suppression of energy-sensing and autophagy signaling pathways^34,35^ (Fig. 3a, b). UA treatment reversed this maladaptive signaling profile, as evidenced by increased AMPK phosphorylation and suppression of mTOR activity. Consistently, phosphorylation of ULK1, a critical initiator of autophagy downstream of AMPK, was restored following UA treatment (Fig. 3a, b). To determine whether these signaling changes translated into functional recovery, we assessed mitochondrial respiration using Seahorse XF analysis. HFpEF stress significantly reduced basal oxygen consumption, ATP-linked respiration, and maximal respiratory capacity. By contrast, UA treatment markedly improved these parameters, restoring both basal and maximal OCR and enhancing spare respiratory capacity (Fig. 3c).

Next, we directly evaluated mitophagic flux using the mt-Keima reporter system in CMs. Under HFpEF-like stress conditions (palmitate + L-NAME), cells predominantly exhibited green fluorescence, indicative of impaired mitophagy. UA treatment significantly increased red fluorescence and the red-to-green ratio, reflecting enhanced mitochondrial delivery to lysosomes (Fig. 3d). We further examined canonical mitophagy markers in cardiac tissue. HFpEF hearts showed reduced expression of PINK1 and Parkin, along with accumulation of p62 and increased LC3-II levels, indicating impaired autophagic flux. UA treatment restored PINK1 and Parkin expression, promoted LC3-II turnover, and facilitated p62 clearance, consistent with reactivation of mitophagy^36–38^ (Fig. 3e, f).

At the ultrastructural level, transmission electron microscopy revealed severe mitochondrial damage in HFpEF hearts, including fragmentation, swelling, and disrupted cristae architecture. UA treatment preserved mitochondrial integrity, maintained cristae organization, and reduced mitochondrial swelling (Fig. 3g). In addition to mitophagy impairment, HFpEF hearts exhibited dysregulated mitochondrial dynamics, characterized by reduced expression of the fusion protein MFN1 and increased levels of the fission regulator DRP1. UA treatment restored MFN1 abundance and normalized DRP1 expression, suggesting improved mitochondrial network homeostasis (Fig. 3h). Collectively, these findings demonstrate that UA restores MQC in HFpEF by activating the AMPK–mTOR–ULK1 signaling axis, thereby promoting mitophagic flux and improving mitochondrial function and structural integrity. These data position AMPK activation as an upstream regulatory event linking mitochondrial dysfunction to impaired mitophagy in HFpEF.

### UA treatment attenuates maladaptive transcriptional reprogramming in human cardiomyocytes under HFpEF-like stress

**Fig. 4:**
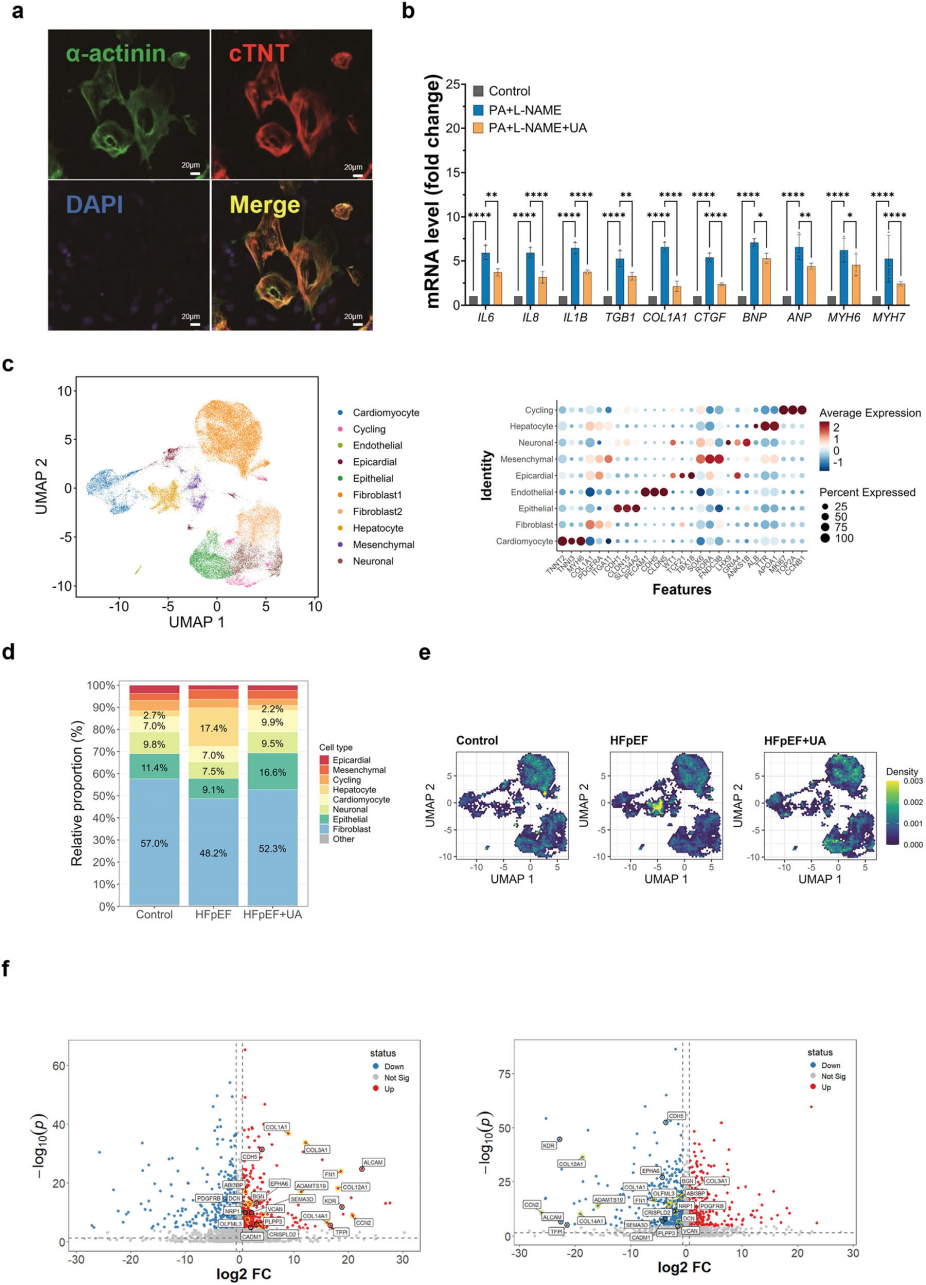
Global snRNA-seq analysis reveals transcriptional landscape changes in human induced pluripotent stem cell-derived cardiomyocytes (hiPSC-CMs) under HFpEF-like stress (HL) and their modulation by urolithin A (UA). **a** Immunofluorescence staining of human induced pluripotent stem cell-derived cardiomyocytes (hiPSC-CMs) for α-actinin, cardiac troponin T (cTnT), and 4′,6-diamidino-2-phenylindole (DAPI) confirmed sarcomeric organization and cardiomyocyte identity (*n* = 3 independent experiments). **b** Quantitative PCR (qPCR) analysis of inflammatory, fibrotic, and hypertrophic marker genes in control, HL, and HL + UA conditions (*n* = 3). **c** snRNA-seq-based cell-state mapping was visualized using UMAP and dot plots of canonical marker genes to annotate major cell populations across experimental groups (*n* = 4). **d** Relative proportions of major cell populations across control, HL, and HL + UA conditions (*n* = 4). **e** UMAP density plots depicting transcriptomic distribution patterns under each experimental condition, shown from left to right as control, HL, and HL + UA (*n* = 4). **f** Volcano plots of differentially expressed genes across the entire hiPSC-CM population comparing HL versus control (left) and HL + UA versus HL (right), with fibrosis-related genes highlighted (*n* = 4). Data are presented as mean ± SEM for qPCR analyses. For in vitro experiments, *n* represents the number of independent experiments (biological replicates), whereas for snRNA-seq analyses, *n* represents the number of biologically independent samples used for sequencing. Exact sample sizes for each analysis are indicated in the corresponding panels. Statistical significance for qPCR analyses was assessed using one-way analysis of variance followed by Dunnett’s multiple comparisons test or the Kruskal–Wallis test followed by Dunn’s post hoc test, as indicated. Other analyses were performed as described in Materials and methods. *P < 0.05, **P < 0.01, ***P < 0.001. FC, fold change; HFpEF, heart failure with preserved ejection fraction; L-NAME, Nω-nitro-L-arginine methyl ester; UMAP, uniform manifold approximation and projection.

To validate these findings in a human system, we utilized hiPSC-CMs, as confirmed by the expression of canonical CM markers (α-actinin and cTnT) (Fig. 4a), and subjected them to HFpEF-like stress conditions (HL; palmitate + L-NAME). Combined metabolic and nitric oxide stress recapitulating HFpEF-like conditions induced the upregulation of inflammatory, fibrotic, and hypertrophic gene expression programs, which were significantly attenuated by UA treatment (Fig. 4b). To further resolve transcriptional remodeling at single-cell resolution, we performed single-nucleus RNA-sequencing (snRNA-seq) on hiPSC-CMs under control, HL, and HL + UA conditions. Unsupervised clustering and UMAP dimensionality revealed distinct transcriptional states across conditions^39–41^ (Fig. 4c). Cell-type annotation identified multiple CM subclusters, including ventricular-like, atrial-like, cycling, and stress-associated populations (Fig. 4c).

Notably, HL stress was associated with the emergence of a transcriptionally distinct subpopulation characterized by the expression of genes typically enriched in hepatocyte-associated programs, including albumin (ALB), transthyretin (TTR), and apolipoprotein A1 (APOA1)^42,43^. However, given the known susceptibility of single-nucleus RNA-sequencing to ambient RNA contamination and transcriptional plasticity under stress conditions, this population is unlikely to represent bona fide hepatocytes but rather reflects a stress-associated, aberrant transcriptional state within CMs. Consistent with this observation, the relative abundance of this hepatocyte-like transcriptional signature was markedly increased under HL conditions and reduced toward control levels following UA treatment (Fig. 4d, e). DGE analysis revealed that HL stress induced coordinated upregulation of profibrotic and proinflammatory gene programs, which were significantly suppressed by UA treatment (Fig. 4f). Collectively, these findings demonstrate that HFpEF-like stress induces maladaptive transcriptional reprogramming and phenotypic plasticity in human CMs, whereas UA mitigates these pathological changes and preserves CM identity and transcriptional homeostasis, thereby preventing maladaptive cellular reprogramming.

### UA treatment prevented the cardiomyocyte fibrogenic transition and restored MQC

**Fig. 5:**
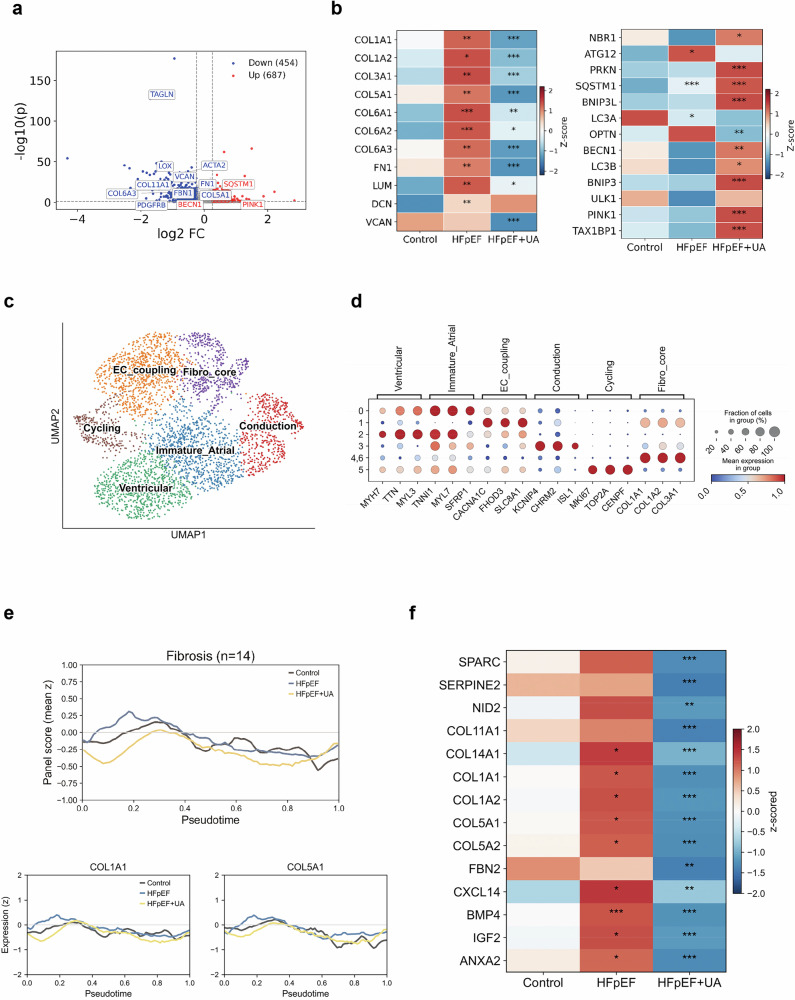
Cardiomyocyte (CM) subset analysis identifies fibrosis-associated transcriptional reprogramming under HFpEF-like stress, which is attenuated by urolithin A (UA). **a** Volcano plot of differentially expressed genes comparing HL + UA and HL, highlighting transcripts significantly upregulated or downregulated following UA treatment. **b** *Z*-score heatmaps of fibrosis-related genes (left) and mitophagy-related genes (right) across control, HL, and HL + UA groups. **c** UMAP embedding of CM subsets reveals major transcriptional modules along a continuous cellular trajectory, including Ventricular, Immature_Atrial, Conduction, EC_coupling, Cycling, and an ECM-enriched Fibro_core module. **d** Dot plot of canonical marker genes defining transcriptional modules along the CM trajectory, with dot size indicating the fraction of cells expressing each gene and color representing scaled mean expression. **e** Pseudotime trajectories of representative genes from a curated fibrosis-associated gene module, including COL1A1 (left) and COL5A1 (right), revealing dynamic expression patterns along the CM state continuum. **f** Heatmap of fibrosis-associated genes along pseudotime, highlighting group-specific expression patterns in control, HL, and HL + UA conditions. Data are presented as mean ± SEM. For snRNA-seq analyses, *n* = 4 biologically independent samples. Exact sample sizes for each analysis are indicated in the corresponding panels. Statistical significance was assessed as described in Materials and methods. *P < 0.05, **P < 0.01, ***P < 0.001. FC, fold change; HFpEF, heart failure with preserved ejection fraction; UMAP, uniform manifold approximation and projection.

To elucidate the transcriptional mechanisms underlying the cardioprotective effects of UA at a single-cell resolution, we performed subcluster analysis and trajectory inference on the CM population from our snRNA-seq dataset. DGE analysis revealed that UA treatment reversed the HFpEF-induced transcriptional signature, characterized by downregulation of profibrotic genes and restoration of mitophagy-related pathways (Fig. 5a, b). To map the continuum of CM transcriptional states, we used pseudotime trajectory analysis^44^. This approach resolved distinct subpopulations, including ventricular, immature atrial, and conduction-type CMs, along with a population highly enriched for ECM gene expression, designated the “Fibro_core” cluster (Fig. 5c, d and Supplementary Fig. 3a). Quantification of cluster proportions revealed that HFpEF stress markedly expanded the Fibro_core population, and UA treatment effectively reduced this population to below control levels (Supplementary Fig. 3b), indicating not only prevention but also active suppression of fibrogenic reprogramming.

Gene set enrichment analysis further revealed that AMPK signaling and MQC-related gene sets exhibited a consistent directional shift in the UA-treated group compared with HFpEF, although these changes did not reach statistical significance (FDR > 0.25) (Supplementary Fig. 3c).

Trajectory analysis further revealed that profibrotic gene expression was progressively induced along early-to-mid pseudotime in HFpEF CMs, consistent with a transition toward a fibrogenic state (Fig. 5e and Supplementary Fig. 3d).

This observation was further supported by fibrosis module scoring, which was significantly elevated in HFpEF and markedly reduced following UA treatment (Supplementary Fig. 3e). In addition, DGE analysis within the Fibro_core cluster showed that UA treatment markedly suppressed key drivers of ECM remodeling and fibrosis, including secreted protein acidic and rich in cysteine (SPARC), serpin family E member 2 (SERPINE2), COL1A1, and bone morphogenetic protein 4 (BMP4)^45–47^ (Fig. 5f). These findings demonstrated that UA treatment prevented the pathological phenotypic plasticity of human CMs by blocking their trajectory toward profibrotic transcriptional states while concurrently restoring MQC programs.

### UA treatment remodels the gut microbiome and reduces systemic ceramide burden in HFpEF

**Fig. 6:**
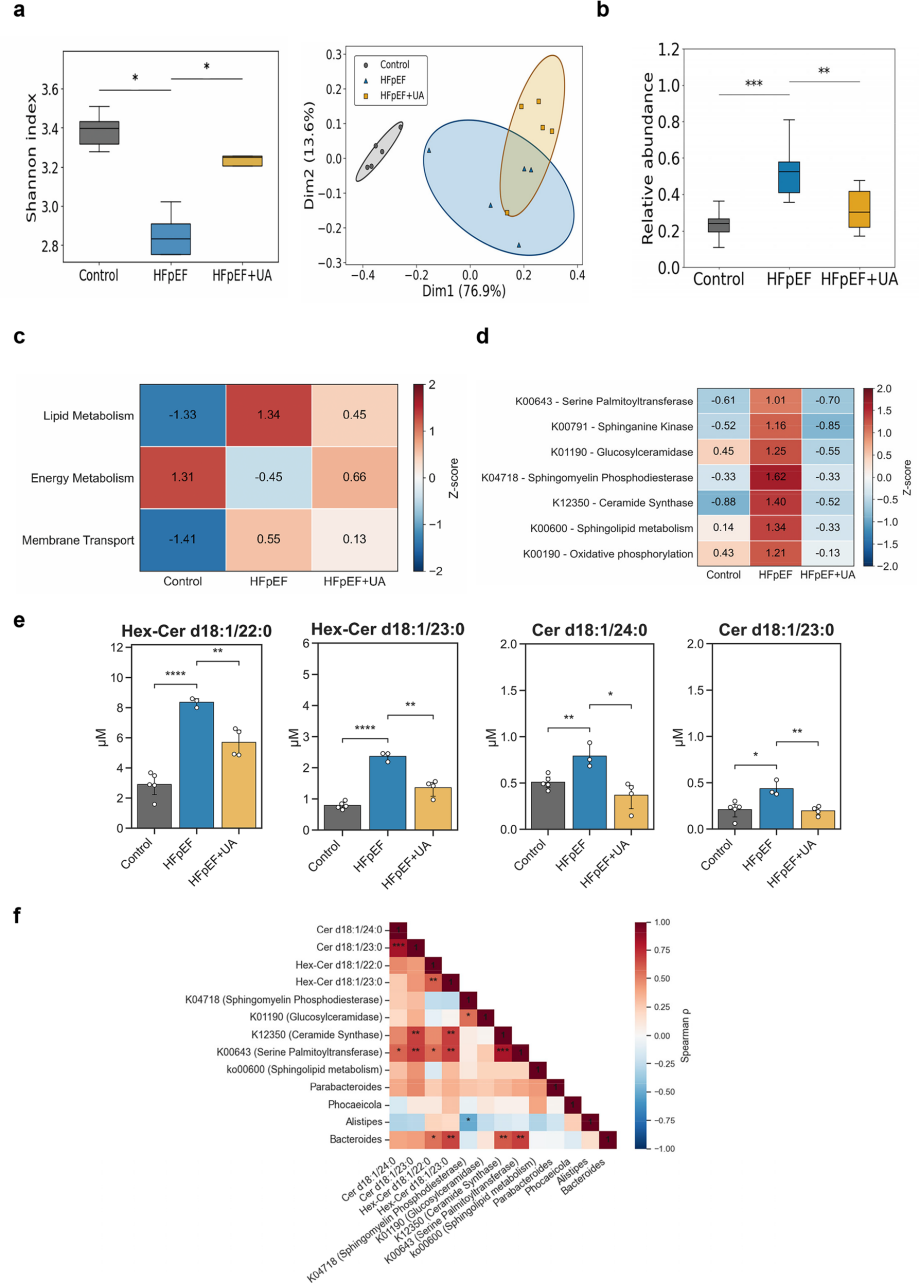
Gut microbiome–ceramide axis is modulated by urolithin A in HFpEF. **a** Microbial diversity analyses showing Shannon α-diversity and Bray–Curtis-based principal coordinates analysis of fecal metagenomic profiles from control, heart failure with preserved ejection fraction (HFpEF), and HFpEF+UA mice (*n* = 5 per group). **b** Relative abundance of microbial taxa implicated in sphingolipid (ceramide) biosynthesis across experimental groups (*n* = 5 per group). **c** *Z*-score heatmap of major Kyoto Encyclopedia of Genes and Genomes (KEGG) functional categories derived from metagenomic functional profiling (*n* = 5 per group). **d** *Z*-score heatmap of KEGG modules related to ceramide and sphingolipid metabolism (*n* = 5 per group). **e** Targeted plasma lipidomic quantification of selected ceramide and hexosyl-ceramide species, shown from left to right as Hex-Cer d18:1/22:0, Hex-Cer d18:1/23:0, Cer d18:1/24:0, and Cer d18:1/23:0 (control, *n* = 5; HFpEF, *n* = 3; HFpEF+UA, *n* = 4). **f** Spearman correlation matrix integrating dominant microbial taxa, ceramide-associated KEGG modules, and circulating ceramide species based on matched samples (control, *n* = 5; HFpEF, *n* = 3; HFpEF+UA, *n* = 4). All data are presented as mean ± SEM. *n* represents the number of biologically independent animals (mice), and exact sample sizes for each analysis are indicated in the corresponding panels. Statistical significance was assessed using one-way analysis of variance followed by Dunnett’s multiple comparisons test for group comparisons, and Spearman correlation for correlation analyses. *P < 0.05, **P < 0.01, ***P < 0.001. UA, urolithin A.

Accumulating evidence has implicated gut dysbiosis in the pathogenesis of HFpEF through the production of cardiotoxic metabolites^39,40^. Given our findings that UA restores MQC and attenuates maladaptive transcriptional remodeling, we next investigated whether these cardioprotective effects are also associated with modulation of the gut microbiome. To this end, we performed shotgun metagenomic sequencing of fecal samples from HFpEF mice. Alpha diversity, assessed by the Shannon index, was significantly reduced in HFpEF and restored by UA treatment (Fig. 6a). Principal coordinate analysis based on Bray–Curtis dissimilarity revealed distinct clustering of microbial communities across groups (Fig. 6a and Supplementary Fig. 2a). The Firmicutes/Bacteroidetes ratio, a hallmark of metabolic dysbiosis^41^, was elevated in HFpEF and normalized by UA treatment (Supplementary Fig. 2b). At the genus level, HFpEF was characterized by the expansion of taxa previously associated with ceramide biosynthesis, including Bacteroides, Parabacteroides, Alistipes, and Phocaeicola, all of which were significantly increased in HFpEF mice^39–41^ (Fig. 6b and Supplementary Fig. 2c). UA treatment suppressed the abundance of these ceramide-associated genera, restoring them toward control levels (Fig. 6b).

Functional profiling using Kyoto Encyclopedia of Genes and Genomes revealed enrichment of lipid metabolism pathways, particularly sphingolipid biosynthesis, in HFpEF microbiomes (Fig. 6c, d). Genes encoding key enzymes in ceramide synthesis including serine palmitoyltransferase (K00643), sphingomyelin phosphodiesterase (K04718), and ceramide synthase (K12350) were upregulated in HFpEF and suppressed following UA treatment (Fig. 6d). Consistent with these microbial alterations, targeted plasma lipidomics revealed elevated levels of ceramides (Cer d18:1/23:0 and Cer d18:1/24:0) and hexosylceramides (HexCer d18:1/22:0 and HexCer d18:1/23:0) in HFpEF mice, which were significantly reduced by UA treatment^47,48^ (Fig. 6e and Supplementary Fig. 2d). Integrative correlation analysis demonstrated positive associations among the abundance of ceramide-associated microbiota, expression of microbial genes involved in sphingolipid metabolism, and circulating ceramide levels (Fig. 6f).

**Fig. 7:**
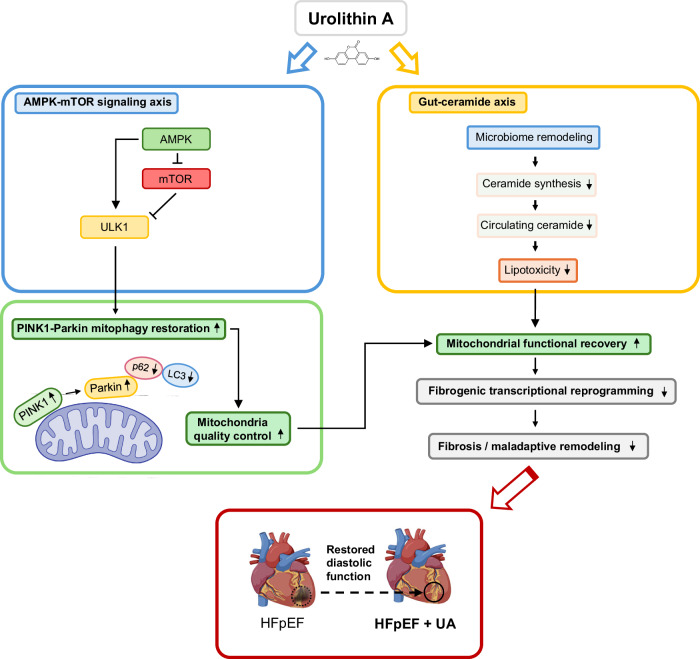
Proposed mechanism of urolithin A-mediated cardioprotection in HFpEF. Schematic illustration of the integrated mechanisms by which urolithin A (UA) ameliorates heart failure with preserved ejection fraction (HFpEF). UA enhances mitochondrial quality control by activating AMPK signaling while inhibiting mTOR, thereby promoting ULK1-dependent mitophagy and restoring impaired mitophagic flux, leading to improved mitochondrial function. In parallel, UA modulates the gut microbiome–ceramide axis, reducing circulating ceramides and lipotoxic stress. Collectively, these coordinated effects attenuate fibrogenic transcriptional reprogramming and cardiac remodeling in HFpEF.

Collectively, these findings indicate that UA treatment remodels the gut microbiome and suppresses microbial sphingolipid biosynthesis, thereby reducing systemic ceramide burden. This microbiome–ceramide axis may act in concert with MQC restoration to mediate the cardioprotective effects of UA in HFpEF. To provide an integrated overview of these findings, we generated a schematic model summarizing the proposed mechanisms by which UA ameliorates HFpEF (Fig. 7), incorporating AMPK signaling, mitophagy restoration, mitochondrial functional recovery, gut microbiome remodeling, ceramide reduction, and suppression of fibrogenic transcriptional reprogramming.

## Discussion

Cardiovascular diseases remain the leading global cause of morbidity and mortality, with HF representing a growing burden^48^. HFpEF represents a major therapeutic challenge largely because it is not an isolated cardiac condition but a heterogeneous syndrome driven by multiple systemic comorbidities, including obesity, hypertension, diabetes, chronic kidney disease, and atrial fibrillation^49^. Accumulating evidence indicates that mitochondrial dysfunction is a central mechanistic link between systemic metabolic perturbations and CM pathology in HFpEF^6,50^. Therapeutic strategies targeting MQC may address the fundamental defects in myocardial energetics and contractile function that underlie this syndrome^51^.

We demonstrated that UA administration ameliorated HFpEF pathology in a two-hit mouse model. UA treatment improved the cardiac structure and function, reduced the left ventricular mass, improved diastolic parameters, and decreased interstitial fibrosis and pulmonary congestion (Fig. 2). We also found that UA treatment restored the phosphorylation of AMPK and its downstream targets (ULK1) while suppressing the elevated mTOR activity observed in HFpEF hearts. This signaling shift re-established the PINK1-Parkin-mediated mitophagic flux, which promoted the clearance of damaged mitochondria and preserved the cristae architecture (Fig. 3 and Supplementary Fig. 1). These findings extend previous observations of the beneficial effects of UA in skeletal muscles and aging models^11,33^, suggesting that its mitophagy-enhancing properties are operational in the stressed myocardium.

Importantly, the precise hierarchical positioning of AMPK activation in this context remains to be fully defined. AMPK is a central metabolic sensor that responds to changes in cellular energy status, and UA has been reported to improve mitochondrial bioenergetics and quality control. Therefore, AMPK activation in our model may arise, at least in part, secondary to improved mitochondrial function. In addition, UA-induced reductions in circulating ceramides may further alleviate metabolic stress, thereby contributing to AMPK activation. These observations suggest that AMPK activation is unlikely to represent a single direct upstream effect of UA, but rather reflects an integrated response to improvements in cellular and systemic metabolic states. Future studies using pharmacological or genetic inhibition of AMPK will be required to establish its causal role in UA-mediated cardioprotection.

A notable finding of our study was the systemic impact of UA on the gut–heart metabolic axis. UA treatment modulated the gut microbiome composition and reduced the abundance of genera, including *Bacteroides* and *Parabacteroides* (Fig. 6 and Supplementary Fig. 2). Metagenomic analysis revealed the downregulation of bacterial genes encoding enzymes involved in de novo ceramide biosynthesis. This microbial shift was correlated with reduced circulating ceramide levels. Given that ceramides promote mitochondrial dysfunction, inflammasome activation, and fibroblast differentiation^52^^,53^, a UA-induced decline in systemic ceramides may interrupt a lipid-driven pathogenic feedback loop, complementing the direct myocardial effects. However, it should be noted that the observed associations among microbiome remodeling, ceramide reduction, and cardiac improvement are correlative in nature. We did not assess the effects of UA on the gut microbiome in control animals, and therefore cannot determine whether microbiome remodeling acts as a primary driver of UA-mediated cardioprotection or occurs as a secondary consequence of improved systemic metabolism. Future studies incorporating germ-free models, microbiome depletion, or fecal microbiota transplantation will be required to establish causal relationships within the gut–ceramide–cardiac axis.

Our snRNA-seq analysis of human iPSC-CMs provided insights into the cellular responses to metabolic stress and UA treatment. Under HFpEF-like conditions, UA treatment attenuated pathological transcriptional programs, reducing the expression of profibrotic markers while maintaining the expression of genes associated with normal CM function and MQC (Figs. 4 and 5). Notably, we identified a CM subpopulation exhibiting hepatocyte-associated gene expression (for example, ALB, TTR, APOA1). This finding should be interpreted with caution. Human iPSC-derived CMs are known to exhibit an immature transcriptional state, which may permit non-lineage-specific gene expression. In addition, metabolic stress may induce transcriptional plasticity, leading to ectopic expression of genes associated with alternative cell types. Technical factors, including ambient RNA contamination or residual doublets, may also contribute. Importantly, similar atypical transcriptional states have been reported in CMs under stress conditions. Therefore, we interpret this cluster as a stress-associated, context-dependent transcriptional state rather than a bona fide hepatocyte lineage. Together, these findings suggest that preservation of MQC may have a central role in maintaining CM transcriptional identity, although the directional relationship between mitochondrial dysfunction and transcriptional reprogramming remains to be fully established.

Our study had several limitations. First, although our two-hit model recapitulated the key features of HFpEF, it represented a relatively short-term intervention in young mice and may not have fully captured the chronic age-related pathophysiology of human HFpEF. Second, although we demonstrated strong correlations among microbiome composition, ceramide levels, and cardiac phenotypes, establishing definitive causal relationships requires additional experimental approaches, such as germ-free models or targeted microbial manipulations. In addition, glucose tolerance and insulin sensitivity were not assessed in this study, limiting our ability to determine the contribution of systemic metabolic changes to the observed cardioprotective effects of UA. Third, although human iPSC-CMs provide human-relevant mechanistic insights, they exhibit a relatively immature phenotype compared with the adult myocardium, potentially limiting translatability.

Despite these limitations, our study provided mechanistic evidence that UA targets multiple interconnected pathways involved in the pathogenesis of HFpEF. By restoring MQC, modulating the gut microbiome composition and systemic ceramide metabolism, and preventing maladaptive CM transcriptional changes, UA addressed several core features of HFpEF pathophysiology in this preclinical model. These findings provide a rationale for the clinical investigation of UA as a potential therapeutic approach for HFpEF, although substantial additional work is required to establish its safety, efficacy, and optimal dosing in humans.

## Supplementary information

Supplementary information
