# Supplementary material for: Urolithin A activates mitophagy via the AMPK–mTOR axis and modulates the gut–ceramide axis to ameliorate cardiac remodeling in HFpEF
Source: Exp Mol Med. 2026 Jul 10;58(7):2325–38. doi: 10.1038/s12276-026-01776-2 (PMC13434734; doi:10.1038/s12276-026-01776-2)
Supplement: Supplementary file 1 [file 12276_2026_1776_MOESM1_ESM.pdf]

Supplementary Table 1. Primer sequences used for RT-PCR

Gene	Species	Direction	Sequence (5′ → 3′)
Mouse primers			
Tgf beta-1	Mouse	Forward	CCTGTCCAAACTAAGGC
	Mouse	Reverse	GGTTTTCTCATAGATGGCG
Col1a1	Mouse	Forward	GAGCGGAGAGTACTGGATCG
	Mouse	Reverse	GCTTCTTTTCCTTGGGGTTC
Ctgf	Mouse	Forward	CAAAGCAGCTGCAAATACCA
	Mouse	Reverse	GGCCAAATGTGTCTTCCAGT
Myh7	Mouse	Forward	CACCGCTCACCTTCACCCG
	Mouse	Reverse	TTCCACCTAAAGGGCTGTTG
Bnp	Mouse	Forward	TCCATCAGAGGGGTCACAC
	Mouse	Reverse	GCCTTGTGAAGGGGTGATTA
Scd1	Mouse	Forward	CCTGTACGCTGGTGCTGAAT
	Mouse	Reverse	CACGTTGGGTCGATGAAGAA
Rab7	Mouse	Forward	ACTGCCCCCAACACTTTCAA
	Mouse	Reverse	CTTGGCACTGGTCTCGAAGT
Pink1	Mouse	Forward	CACACTGTTCCTCGTTATGAAGA
	Mouse	Reverse	CTTGAGATCCCGATGGGCAAT
Mfn1	Mouse	Forward	ATCACTGCAATCTTCGGCCA
	Mouse	Reverse	AGCAGTTGGTTGTGTGACCA
Atg9a	Mouse	Forward	CGCTGTGGACAAAGTGATGA
	Mouse	Reverse	GCTGGTAGATGGTGTTGAGG
Human primers			

Gene	Species	Direction	Sequence (5′ → 3′)
IL6	Human	Forward	AGTAGTGAGGAACAAGCCAGAG
	Human	Reverse	TACATTTGCCGAAGAGCC
IL8	Human	Forward	GAGAGTGATTGAGAGTGGACCAC
	Human	Reverse	CACAACCCTCTGCACCCAGTTT
IL1B	Human	Forward	CCACAGACCTTCCAGGAGAATG
	Human	Reverse	GTGCAGTTCAGTGATCGTACAGG
TGFB1	Human	Forward	TCGACATGGAGCTGGTGAAG
	Human	Reverse	GGTTTCCACCATTAGCACGC
COL1A1	Human	Forward	GAGGGCCAAGACGAAGACATC
	Human	Reverse	CAGATCACGTCATCGCACAAC
CTGF	Human	Forward	CAAGGGCCTCTTCTGTGACT
	Human	Reverse	CCGTCGGTACATACTCCACA
BNP	Human	Forward	TCAGCCTCGGACTTGGAAAC
	Human	Reverse	CTTCCAGACACCTGTGGGAC
ANP	Human	Forward	GAACCAGAGGGGAGAGACAGAG
	Human	Reverse	CCCTCAGCTTGCTTTTTAGGAG
MYH6	Human	Forward	GGAAGACAAGGTCAACAGCCTG
	Human	Reverse	TCCAGTTTCCGCTTTGCTCGCT
MYH7	Human	Forward	AGACTGTCGTGGGCTTGTATCAG
	Human	Reverse	GCCTTTGCCCTTCTCAATAGG

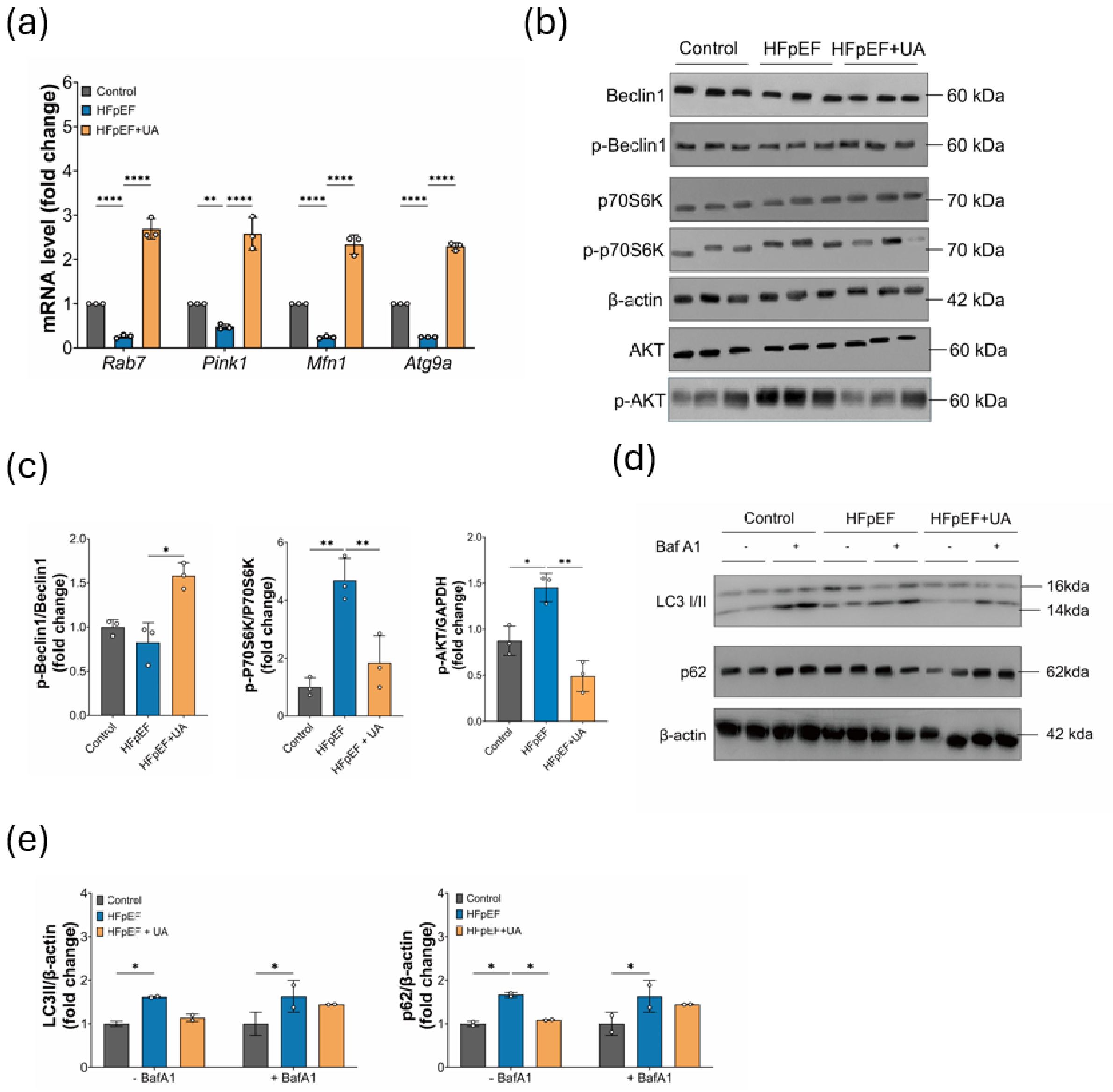

Supplementary Figure 1. **(a)** Quantitative PCR analysis of autophagy- and mitophagy-related genes in cardiac tissue from Control, HFpEF, and HFpEF+UA mice. **(b)** Immunoblot analysis of phospho-Beclin-1/Beclin-1, phospho-p70S6K/p70S6K, phospho-AKT/AKT and in heart lysates; β-actin was used as a loading control. **(c)** Densitometric quantification of phospho-mTOR/mTOR, phospho-AKT/AKT, and phospho-AMPKα/AMPKα ratios. **(d)** Immunoblot analysis of autophagy initiation–related proteins, including Beclin1, ULK1, p70S6K; β-actin was used as a loading control. **(e)** Densitometric quantification of phospho-Beclin1/Beclin1, phospho-ULK1/ULK1, and phospho-

p70S6K/p70S6K ratios. Data are presented as mean ± SEM. Statistical significance was assessed as described in the Methods. *P<0.05, **P<0.01, ***P<0.001, ****P<0.0001.

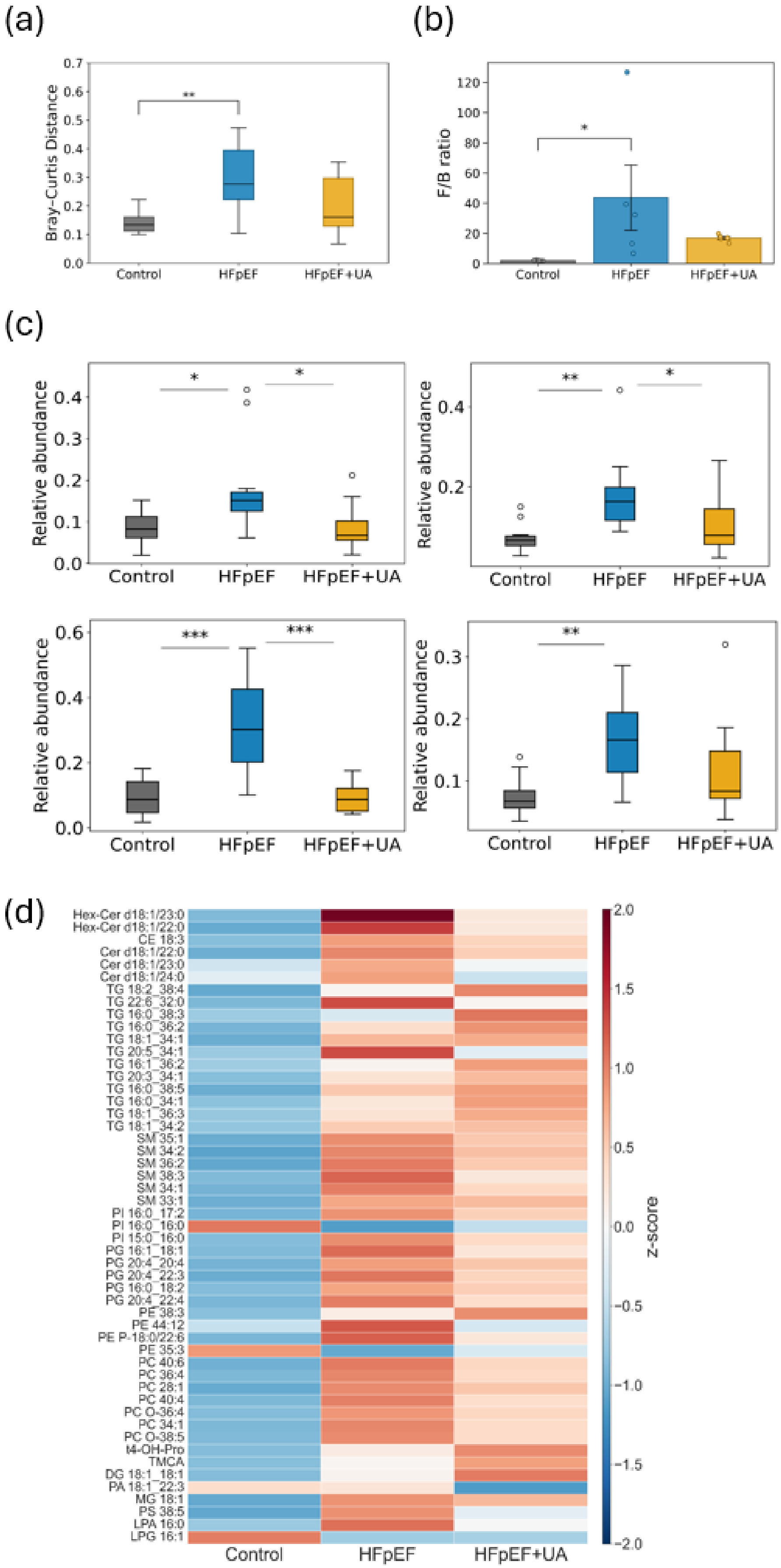

(a)
(b)
Bray-Curtis Distance
0.0
0.1
0.2
0.3
0.4
0.5
0.6
0.7
**
Control
HFpEF
HFpEF+UA
F/B ratio
0
20
40
60
80
100
120
*
Control
HFpEF
HFpEF+UA
(c)
Relative abundance
0.0
0.1
0.2
0.3
0.4
*
*
Control
HFpEF
HFpEF+UA
Relative abundance
0.2
0.4
**
*
Control
HFpEF
HFpEF+UA
Relative abundance
0.0
0.2
0.4
0.6
***
***
Control
HFpEF
HFpEF+UA
Relative abundance
0.1
0.2
0.3
**
Control
HFpEF
HFpEF+UA
(d)
Hex-Cer d18:1/23:0
Hex-Cer d18:1/22:0
CE 18:3
Cer d18:1/22:0
Cer d18:1/23:0
Cer d18:1/24:0
TG 18:2_38:4
TG 22:6_32:0
TG 16:0_38:3
TG 16:0_36:2
TG 18:1_34:1
TG 20:5_34:1
TG 16:1_36:2
TG 20:3_34:1
TG 16:0_38:5
TG 16:0_34:1
TG 18:1_36:3
TG 18:1_34:2
SM 35:1
SM 34:2
SM 36:2
SM 38:3
SM 34:1
SM 33:1
PI 16:0_17:2
PI 16:0_16:0
PI 15:0_16:0
PG 16:1_18:1
PG 20:4_20:4
PG 20:4_22:3
PG 16:0_18:2
PG 20:4_22:4
PE 38:3
PE 44:12
PE P-18:0/22:6
PE 35:3
PC 40:6
PC 36:4
PC 28:1
PC 40:4
PC O-36:4
PC 34:1
PC O-38:5
t4-OH-Pro
TMCA
DG 18:1_18:1
PA 18:1_22:3
MG 18:1
PS 38:5
LPA 16:0
LPG 16:1
Control
HFpEF
HFpEF+UA
2.0
1.5
1.0
0.5
0.0
−0.5
−1.0
−1.5
−2.0
z-score

Supplementary Figure 2. Gut microbiome composition and lipidomic features associated with HFpEF and UA treatment. (a) Bray–Curtis dissimilarity analysis comparing fecal microbial community composition among Control, HFpEF, and HFpEF+UA groups. **(b)** Firmicutes/Bacteroidetes (F/B) ratio calculated from metagenomic profiles. **(c)** Relative abundance of selected ceramide-producing bacterial genera across experimental groups, shown from left to right as Bacteroides, Parabacteroides, Phocaeicola, and Alistipes. **(d)** Z-score heatmap of selected lipidomic species across Control, HFpEF, and HFpEF+UA groups. Data are presented as mean ± SEM. Statistical significance as assessed as described in the Methods. *P<0.05, **P<0.01, ***P<0.001.

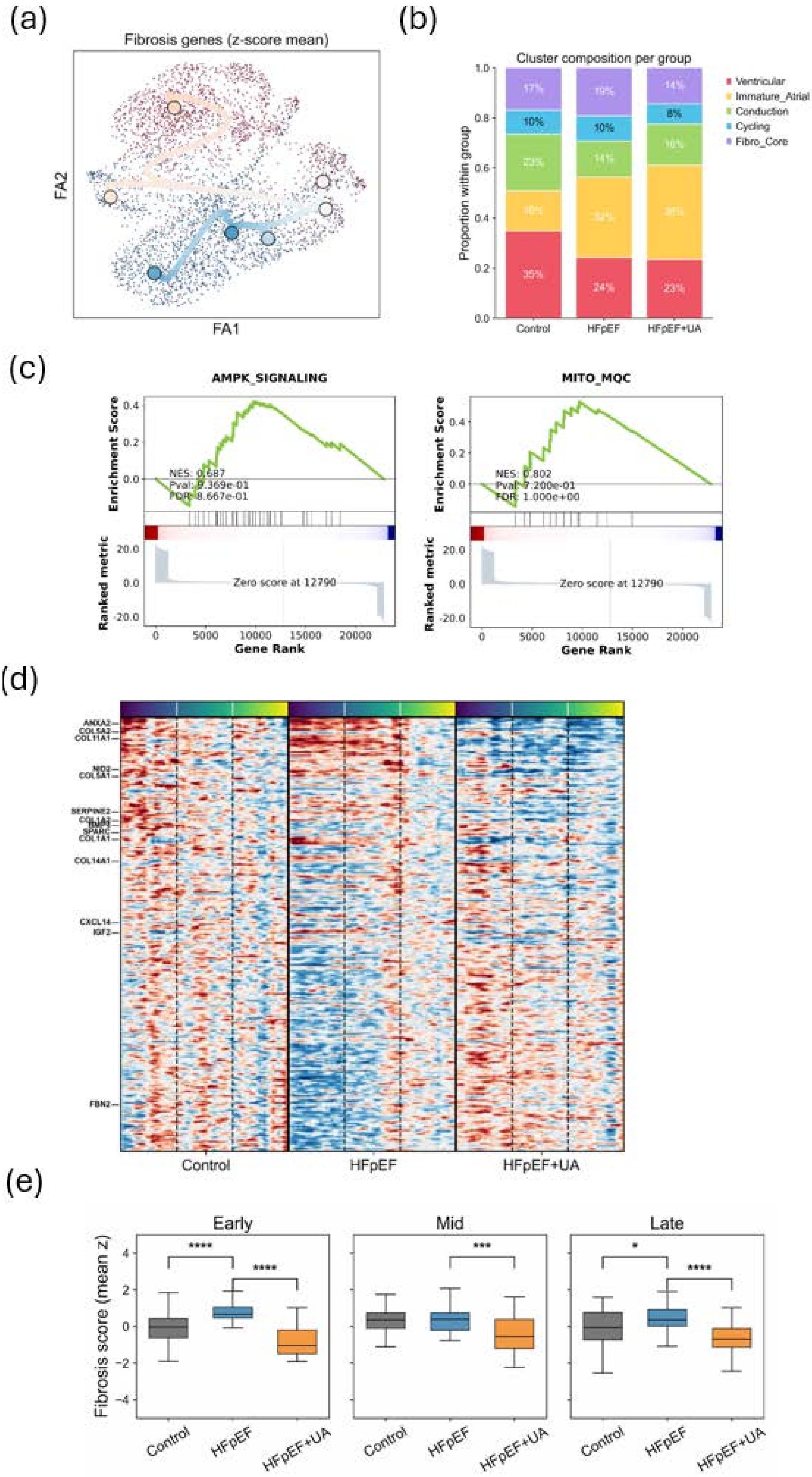
(a)
Fibrosis genes (z-score mean)
FA2
FA1
(b)
Cluster composition per group
Proportion within group
1.0
0.8
0.6
0.4
0.2
0.0
Ventricular
Immature_Atrial
Conduction
Cycling
Fibro_Core
17%
10%
23%
35%
10%
24%
8%
23%
14%
Control
HFpEF
HFpEF+UA
(c)
AMPK_SIGNALING
MITO_MQC
Enrichment Score
NES: 0.687
Pval: 9.369e-01
FDR: 8.667e-01
NES: 0.802
Pval: 7.200e-01
FDR: 1.000e+00
Ranked metric
Zero score at 12790
Gene Rank
0
5000
10000
15000
20000
(d)
ANXA2
COL5A2
COL11A1
COL5A1
SERPINE2
SPARC
COL1A1
COL14A1
CXCL14
IGF2
FBN2
Control
HFpEF
HFpEF+UA
(e)
Early
Mid
Late
Fibrosis score (mean z)
4
2
0
-2
-4
****
***
*
Control
HFpEF
HFpEF+UA

Supplementary Figure 3. Fibrosis-associated transcriptional programs and cardiomyocyte state dynamics. (a) ForceAtlas2 (FA1–FA2) embedding of cardiomyocyte nuclei visualizing the mean Z-score expression of a fibrosis-associated gene panel along inferred transcriptional trajectories. **(b)** Relative composition of major cardiomyocyte transcriptional modules across Control, HFpEF, and HFpEF+UA groups. **(c)** Directional GSEA changes in AMPK signaling and mitochondrial quality control with UA treatment **(d)** Heatmap of significantly altered fibrosis-related genes within fibroblast-enriched transcriptional states, showing per-cell scaled expression across Control, HFpEF, and HFpEF+UA cardiomyocytes. **(e)** Pseudotime dynamics of the fibrosis gene module, displayed as mean Z-score panel scores across Early, Mid, and Late pseudotime phases in Control, HFpEF, and HFpEF+UA cardiomyocytes.
